# Development of value‐added nutritious crackers with high antidiabetic properties from blends of *Acha* (*Digitaria exilis*) and blanched Pigeon pea (*Cajanus cajan*)

**DOI:** 10.1002/fsn3.748

**Published:** 2018-08-13

**Authors:** Aderonke Ibidunni Olagunju, Olufunmilayo Sade Omoba, Victor Ndigwe Enujiugha, Rotimi Emmanuel Aluko

**Affiliations:** ^1^ Department of Food Science and Technology Federal University of Technology Akure Ondo State Nigeria; ^2^ Department of Food and Human Nutritional Sciences University of Manitoba Winnipeg Manitoba Canada

**Keywords:** *acha*–pigeon pea crackers, antidiabetic properties, antioxidant properties, nutritional composition

## Abstract

Pigeon pea was treated by blanching and used to supplement *acha* flour for the development of functional cracker biscuits. The flour ratios for *acha* and pigeon pea were 100:0 (ACC), 80:20 (APC1), and 70:30 (APC2), respectively. The developed cracker biscuits were evaluated for chemical acid compositions, antioxidant, as well as antidiabetic properties. Protein contents of the formulated crackers increased with increase in supplementation with pigeon pea flour. The antinutrient content of the formulated snack was low hence may not adversely affect nutrient bioavailability. Glutamic and aspartic acids were the predominant amino acids while methionine and lysine significantly increased as a result of supplementation with pigeon pea flour. The biscuit exhibited good antioxidant properties indicated by its strong ability to scavenge hydroxyl, superoxide, DPPH radicals, and reduced Fe^3+^ to Fe^2+^. The formulated snack especially APC2 possessed low glycemic index (47.95%) and significantly inhibited the key digestive enzymes (α‐amylase and α‐glucosidase). All parameters evaluated indicated that APC2 could serve as a functional snack in the management of hyperglycemia (diabetes) and prevention of associated degenerative diseases.

## 1 INTRODUCTION

Cereals and legumes play important roles in human nutrition. Recent reports have shown that they contain constituents with demonstrated health benefits for humans, such as antioxidant and antidisease factors (Durazzo, Casale, Melini, Maian, & Acquistucci, 2015; Gani, Wani, Masoodi, & Hameed, 2012; Shahwar, Bhat, Ansari, Chaudhary, & Aslam, 2017). Legume proteins play a vital role in complementing cereals in the production of ready to eat snacks. Their use as functional ingredients in food formulations is receiving increased attention (Vaz Patto et al., 2015). Studies have shown that consumption of legumes contributes several physiological and health benefits such as prevention of cardiovascular diseases, obesity, and diabetes. Diabetes consists of a group of metabolic disorder characterized by elevated blood glucose, either because insulin production is inadequate or because the body's cells do not respond properly, or both. The number of global diabetes cases was 171 million in 2010 and is predicted to rise to 366 million by 2030 (Si et al., 2010). Its increasing worldwide incidence constitutes a global health burden. Elevated levels of glucose in human body have been linked to the generation of reactive oxygen species (ROS) and alteration of endogenous antioxidants (Ademiluyi & Oboh, 2012). Therefore, maintenance of body antioxidant status is important in the management of type 2 diabetes mellitus. The key enzymes involved in the breakdown of complex carbohydrates are salivary α‐amylase, pancreatic α‐amylase, and intestinal α‐glucosidase. α‐amylase is an endo‐acting enzyme which catalyzes the hydrolysis of α‐D‐glycosidic linkages of starch, amylose, amylopectin, and various maltodextrins. It is involved in the breakdown of long‐chain carbohydrates to maltose while α‐glucosidase breaks down starch and disaccharides to glucose. The inhibition of these key digestive enzymes leads to decreased meal‐derived glucose absorption. α‐amylase inhibitors decrease the high glucose level that can occur after a meal by slowing the speed at which α‐amylase converts starch to simple sugars. On the other hand, α‐glucosidase inhibitors prevent the digestion of carbohydrates by these enzymes. Currently, the constraint to the use of synthetic inhibitors such as acarbose, voglibose, and miglitol is the exorbitant prices and the associated clinical side effects such as hypoglycemia, weight gain (Thulé & Umpierrez, 2014), and the non‐tolerance by some patients (Dujic et al., 2015). Inhibitors derived from natural products do not have side effects, and the therapies are well tolerated. Hence, the increase in the search for food derived natural inhibitors of the key enzymes. Foods with high carbohydrate and dietary fiber contents, especially cereals, have been reported to allow withdrawal of oral hypoglycemic agents leading to a reduction in the insulin dose in diabetic patients (Kutos, Golob, Kac, & Plestenjak, 2003). Proteinaceous α‐amylase inhibitors are found in cereals and legumes (Sivakumar, Mohan, Franco, & Thayumanavan, 2006). Legumes are regularly consumed, and they form major components of many food preparations, especially because they have been recommended for the management of diabetes and some cardiovascular conditions owing mainly to their high dietary fiber and low sodium contents (Foster‐Powell & Miller, 1995; Madar & Stark, 2002). Legumes are commonly used to supplement cereal flours as they increase the nutritional composition and functionality of the resulting products. Biscuits are the largest category of snack items among baked products. Biscuits constitute the easiest means to access the entire population of a country attributable to their eating convenience, low production cost, and wide consumption pattern. *Acha* (*Digitaria exilis*) is one of the indigenous grains in West Africa. It is a unique cereal with relatively high sulfur amino acid (methionine and cystine) content, and it has been shown to have a low glycemic index (Balde, Besancon, & Sidibe, 2008). Moreover, *acha* has been exploited in the treatment and management of diabetes (Jideani & Jideani, 2011). Pigeon pea (*Cajanus cajan*) on the other hand is an underutilized legume rich in protein, minerals, and other phytonutrients. This study was carried out to develop ready‐to‐eat snacks (crackers) from the blends of *acha* and pigeon pea flours as well as to evaluate the functionality of crackers from the flour blends and its potential in the management of hyperglycemia.

## 2 MATERIALS AND METHODS

*Acha* (*Digitaria exilis*) was purchased from Minna central market, Minna, Niger State, Nigeria, and pigeon pea (*Cajanus cajan*) seeds (TCc‐AO/TB78‐9) were obtained from the Gene Bank of the International Institute of Tropical Agriculture, Ibadan, Oyo State, Nigeria. Ammonia, sodium metabisulfite (SMB), lecithin, yeast, and enzymes were obtained from a biscuit company in Lagos, Nigeria. All other reagents used were of analytical grade and purchased from renowned chemical stores in Akure, Ondo State, Nigeria, and Canada. *Acha* and pigeon pea seeds were processed into flours by sorting, dehulling, drying, milling, and sieving according to the methods of Olapade, Aworh, and Oluwole (2011) and Fasoyiro et al. (2010), with slight modifications. The pigeon pea seeds were blanched by soaking in boiling water (100°C) for 3 h prior to dehulling and further processing. Composite flours were made by substituting *acha* flour with 20% and 30% pigeon pea flours. The ratios were generated from a preliminary study which ascertained the ratios were the best in terms of increased protein content, reduced antinutrient content, and overall sensory acceptability.

The cracker biscuits were produced as described by Han, Janz, and Gerlat (2010), with slight modification. Creaming was carried out to premix margarine, sweetner (honey), lecithin, and ammonia. Flour (*acha*, composite flour) and SMB were mixed together and hydrated with water. It was subsequently added to the cream and mixed for about 5 min to obtain a hard‐extensive dough texture. The dough was allowed to ferment for 3 h and thereafter kneaded to a thickness of 1 mm. It was cut using a circularly shaped biscuit cutter, and docking was carried out to create air spaces. The baking was carried out at 150–200°C for 15 min.

### 2.1 Determination of chemical composition

The moisture content (hot air oven method) and fat content (using soxhlet extraction method) were determined as described by Pearson (1976) while ash and protein were determined using AOAC (2012) methods. The carbohydrate contents were calculated by difference.

### 2.2 Determination of amino acid composition

The amino acid profiles were determined using the HPLC Pico‐Tag system according to the method previously described by Bidlingmeyer, Cohen, and Tarvin (1984) after samples were digested with 6 M HCl for 24 h. The cysteine and methionine contents were determined after performic acid oxidation (Gehrke, Wall, Absheer, Kaiser, & Zumwalt, 1985), and the tryptophan content was determined after alkaline hydrolysis (Landry & Delhaye, 1992).

### 2.3 Determination of antioxidant properties of the biscuit extract

Aqueous extract of the biscuit was obtained by milling and hydrating 10 g of the milled biscuit in 100 ml of double distilled water for 24 h using a stirred plate. The suspension was thereafter centrifuged at 9000 *g* for 20 min, and the supernatant was filtered and stored at 4°C for further analyses.

#### 2.3.1 Determination of DPPH radical scavenging activity (DRSA)

The scavenging activity of the biscuit extract against the DPPH radical was determined using the method described by Girgih, Udenigwe, Li, Adebiyi, and Aluko (2011). Samples were mixed with 0.1 M sodium phosphate buffer, pH 7.0 containing 1% (v/v) Triton‐X. DPPH was dissolved in methanol to a final concentration of 100 μM. A 100‐μl aliquot of each sample was mixed with 100 μl of the DPPH radical solution in a 96‐well plate and incubated at room temperature in the dark for 30 min. The buffer was used in the blank assay while reduced glutathione (GSH) served as the positive control. Absorbance was measured at 517 nm using a microplate reader and the percentage DPPH radical scavenging activity was determined using the following equation:

$$\begin{array}{cc} & \text{DPPH radical scavenging activity}\;=\;\\ & \;\frac{\text{Absorbance (blank) ‐ Absorbance (sample)}}{\text{Absorbance (blank)}}\times 100 \end{array}$$

#### 2.3.2 Determination of superoxide radical scavenging activity (SRSA)

The method described by Xie, Huang, Xu, and Jin (2008) was used to determine SRSA. Samples were each diluted in 50 mM Tris–HCl buffer, pH 8.3 containing 1 mM EDTA and 80 μl was transferred into a clear bottom microplate well; 80 μl of buffer was added to the blank well. This was followed by addition of 40 μl 1.5 mM pyrogallol (dissolved in 10 mM HCl) into each well in the dark, and the change in the rate of reaction was measured immediately at room temperature over a period of 4 min using a microplate reader at a wavelength of 420 nm. The superoxide scavenging activity was calculated using the following equation:

$$\begin{array}{cc} & \text{Superoxide scavenging activity}\;(\%)=\\ & \;\frac{\Delta A/\;\min\;(\text{Blank})-\Delta A/\;min\;(\text{sample})}{\Delta A/\;\min\;(\text{blank})}\times 100 \end{array}$$

#### 2.3.3 Determination of hydroxyl radical scavenging activity (HRSA)

The hydroxyl radical scavenging assay was modified based on a method described by Girgih et al. (2011). An aliquot (50 μl) of sample or GSH or buffer (control) was first added to a clear, flat bottom 96‐well plate followed by additions of 50 μl of 1, 10‐phenanthroline, and 50 μl of FeSO_4_. To initiate reaction in the wells, 50 μl of hydrogen peroxide (H_2_O_2_) solution was added to the mixture, which was then covered and incubated at 37°C for 1 h with shaking. Thereafter, the absorbance of the mixtures was measured at 536 nm every 10 min for a period of 1 h. The hydroxyl radical scavenging activity was calculated as follows based on change in absorbance (Δ*A*):

$$\begin{array}{cc} & \text{Hydroxyl radical scavenging activity}=\\ & \;\frac{\Delta A/\;\min\;(\text{control})-\Delta A/\;min\;(\text{sample})}{\Delta A/\;\min\;(\text{control})}\times 100 \end{array}$$

#### 2.3.4 Determination of ferric‐reducing antioxidant property (FRAP)

The reducing power of the biscuit samples was determined according to the modified method of Benzie and Strain (1996). Sample or GSH was dissolved in 0.3 M acetate buffer (pH 6.6). The FRAP reagent was prepared by mixing 0.3 M acetate buffer with 10 mM TPTZ at pH 3.6 in 40 mM HCl and 20 mM FeCl_3_. 6H_2_O, at the ratio of 5:1:1 (v/v/v), respectively. Solutions of FeSO_4_·7H_2_O in the range of concentrations of 0.0625–1.0 μmol/μl were used for the calibration. The samples (40 μl) were placed into a clear 96‐well plate, and 200 μL of the FRAP reagent heated to 37°C was added. The reaction was carried out at 37°C, and the absorbance was read at 593 nm. The results were expressed in mmol of Fe^2+^ per mg of sample based on the calibration curve.

### 2.4 Determination of antidiabetic properties

#### 2.4.1 Determination of α‐amylase inhibition

The inhibitory activity on α‐amylase was determined according to a modified version of an assay from the Worthington Enzyme Manual (Worthington, 1993). A total of 100 μl of sample and 100 μl of 20 mmol/L sodium phosphate buffer (pH 6.9) containing a‐amylase solution (1 mg/ml) and 6 mmol/L NaCl were incubated at 25°C for 10 min. A volume of 100 μl of 1% starch solution in 20 mmol/L sodium phosphate buffer (pH 6.9, containing 6 mmol/L NaCl) was subsequently added to the sample. The reaction mixtures were then incubated at 25°C for 10 min. The reaction was terminated by adding 200 μL of dinitrosalicylic acid reagent, followed by incubation in a boiling water bath for 5 min. The sample was then cooled to room temperature and added to 3 ml of distilled water. The absorbance of the sample, Control 1 or Control 2 was measured at 540 nm. The Control 1 was a mixture of starch solution and sample without the addition of enzyme, whereas Control 2 was a mixture of starch solution and enzyme without addition sample.

The α‐amylase inhibitory activity was expressed as percent inhibition and calculated using the following equation:

$$\begin{array}{cc} & \text{Inhibition of}\;\alpha -\text{amylase activity}\;(\%)=\\ & \;\frac{\text{Abs (control2)}-[\text{Abs (sample)}-\text{Abs (control1)}]}{\text{Abs (control2)}}\times 100 \end{array}$$

#### 2.4.2 Determination of α‐glucosidase inhibition

α‐Glucosidase assay was carried out according to the method of Kwon, Apostolidis, Kim, and Shetty (2007) with a slight modification. About 300 mg of rat‐intestinal acetone powder was suspended in 9 ml of 0.9% saline (NaCl solution), and the suspension was centrifuged (10,000 *g*, 30 min, 4°C), the resulting supernatant was filtered using 0.45‐μm syringe filter. Samples were hydrated in 0.1 M sodium phosphate buffer (pH 6.9), acarbose served as the positive control. Sample aliquot (50 μl) was pipette into 96‐well clear plate, 50 μl of α‐glucosidase enzyme was added and incubated at 37°C for 10 min. After preincubation, 100 μl of 5 mM ρ‐nitrophenyl‐glucopyranoside solution was added to each well. The reaction mixtures were incubated at 37°C for 30 min, and readings were recovered every 5 min. Before and after incubation, absorbance was read at 405 nm and compared to a control which had 50 μL of buffer solution in place of the extract. The α‐glucosidase inhibitory activity was expressed as inhibition% and was calculated as follows:

$$\begin{array}{cc} & \text{Inhibition of}\;\alpha -\text{glucosidase activity}\;(\%)=\\ & \;\frac{\text{Abs (control)‐Abs (sample)}}{\text{Abs (control)}}\times 100 \end{array}$$

#### 2.4.3 Determination of in vitro starch hydrolysis and estimated glycemic index (eGI)

In vitro starch hydrolysis rate and hydrolysis index were determined according to the method of Gooni, Garcia‐Alonso, and Saura‐Calixto (1997). Samples (50 mg each) were incubated with 1 mg, of pepsin in 10 ml HCl‐KCl buffer (pH 1.5) at 40°C for 60 min in a shaking water bath. The digest was diluted to 25 ml by adding phosphate buffer (pH 6.9), and then, 5 ml of α‐ amylase solution containing 0.005 g of α‐amylase in 10 ml of buffer was added. The samples were incubated at 37°C in a shaking water bath. A known value of 0.1 ml sample was taken from each flask every 30 min from 0 to 3 h and boiled for 15 min to inactivate the enzyme. Sodium acetate buffer (1 ml 0.4 M, pH 4.75) was added and the residual starch digested to glucose by adding 30 ml amyloglucosidase and incubating at 60°C for 45 min. Glucose concentration was determined by adding 200 μl of dinitrosalicylic acid color reagent the reaction mixtures was stopped by placing the tubes in a water bath at 100°C for 5 min and then cooled to room temperature. The reaction mixture was then diluted by adding 5 ml of distilled water, and the mixture was centrifuged. The supernatant was collected and the absorbance measured at 540 nm. The rate of starch digestion was expressed as the percentage of starch hydrolyzed per time. Aliquot (50 mg) of glucose was used as the standard.

$$\text{Hydrolysis Index}\;\left(\%\right)=\frac{\text{AUC (sample)}}{\text{AUC (ref)}}\times 100$$

eGI = 39.71 + 0.549 HI

where GI = Glycemic Index (%); and HI = Hydrolysis Index (%).

### 2.5 Determination of in vitro protein digestibility (IVPD)

The in vitro protein digestibility was estimated using a multienzyme technique described by Hsu, Vavak, Satterlee, and Miller (1977). Sample was ground and dissolved in double distilled water (DDQ). Aliquot (50 ml) of the aqueous protein suspension (6.25 mg/ml) was adjusted to pH 8.0 with 0.1 M NaOH/HCl and stirred on a thermostat stirred plate at 37°C. The multienzyme solution (1.6 mg trypsin, 3.1 mg chymotrypsin, 1.3 mg peptidase/ml) was maintained in an ice bath and adjusted to pH 8.0. Aliquot (5 ml) of the multienzyme solution was added to the protein suspension stirred at 37°C. The pH drop was recorded automatically over a 10‐min period using a recording pH meter and the protein digestibility calculated as shown below:

Y=210.46−18.10x

Y = IVPD (%); *x* = pH of protein suspension after 10 min of multienzyme digestion

### 2.6 Statistical analysis

Data generated was subjected to analysis of variance (ANOVA) using Statistical Package for Social Sciences (SPSS) V. 17.0. The means were separated using Duncan's Multiple Range Test (DMRT) at 95% confidence level.

## 3 RESULTS AND DISCUSSIONS

### 3.1 Chemical composition

The chemical compositions of crackers formulated from different flour blends are presented in Table 1. Ash contents ranged from 1.33% to 3.51%, with ACC showing the highest ash content (3.51%) which suggests that the snack may be a potential source of mineral elements. Crude fiber contents (2.90–7.67%) of the formulated biscuits increased with increase in supplementation, which suggests that pigeon pea flour contributed to the fiber contents of the snacks, being a fiber‐rich legume. Fat contents ranged from 12.83% to 21.39% with the control (BCC) having the highest fat content. There were significant differences (*p *<* *0.05) in the fat contents of the formulated snacks in spite of the fact that equal amount of fat (margarine) were measured for crackers production. The variation could be due to differences in the oil absorption capacities of the different flour blends. Protein contents (10.47–19.18%) increased with increase in percentage substitution with pigeon pea flour from 0% to 30%. Addition of pigeon pea flour in crackers has great potential in overcoming the common menace of protein‐energy malnutrition. Similar result of increased protein content from 5.0% to 14.2% was reported for biscuits from wheat–soybean flour (Banureka & Mahendran, 2009). Chinma, Igbabul, and Omotayo (2012) also reported protein contents of 14.57% and 19.80% for unripe plantain‐defatted sesame cookies (80:20, 70:30, respectively). Similarly, Hanan (2015) reported a 14% protein in chickpea (25%) supplemented biscuit. Increase in protein content could be attributed to the fact that pigeon pea flour contributed to the protein content itself, being a protein‐rich legume similar to other legumes (cowpea, soybean, field pea, etc.). High carbohydrate content (65.42%) was observed for 100% *acha* biscuit (ACC). This could be due to the fact that the biscuit is composed mainly of *acha* cereal which has high carbohydrate content. Carbohydrate contents of formulated biscuits decreased from 65.42% to 54.59% as level of *acha* flour substitution increased. This implies that increased supplementation with legume and reduced percentage of cereal flour resulted in a reduction in carbohydrate contents and consequent increase in protein contents. Supplementation with pigeon pea flour contributed to reduced caloric contents; thus, the biscuit may be useful to consumers with health challenge requiring low carbohydrate content foods.

**Table 1:** Chemical composition of formulated crackers biscuit

	Samples			
Constituents	BCC	ACC	APC1	APC2
Ash (%)	1.78 ± 0.03^b^	3.51 ± 0.01^a^	1.33 ± 0.03^c^	1.63 ± 0.04^b^
Crude fiber (%)	2.90 ± 0.11^c^	2.99 ± 0.07^c^	5.08 ± 0.09^b^	7.67 ± 0.20^a^
Fat (%)	21.39 ± 0.47^a^	12.83 ± 0.30^c^	17.26 ± 0.60^b^	15.22 ± 0.06^b^
Protein (%)	9.32 ± 0.20^d^	10.47 ± 0.02^c^	13.67 ± 1.73^b^	19.18 ± 0.12^a^
Carbohydrate (%)	60.14 ± 0.67^b^	65.42 ± 0.08^a^	59.08 ± 0.05^b^	54.59 ± 0.78^c^
Phytate (mg/100 g)	1.63 ± 0.11^a^	1.36 ± 0.02^b^	0.23 ± 0.06^d^	0.35 ± 0.14^c^
Tannin (mg/100 g)	0.09 ± 0.01^c^	2.78 ± 0.16^a^	2.02 ± 0.09^b^	2.26 ± 0.05^a^
Oxalate (mg/g)	0.09 ± 0.00^c^	0.09 ± 0.01^c^	0.18 ± 0.00^b^	0.27 ± 0.01^a^
TI (mg/g)	0.04 ± 0.01^c^	0.11 ± 0.02^b^	0.24 ± 0.01^a^	0.28 ± 0.03^a^
Calcium (g/100 g)	0.03 ± 0.00^b^	0.03 ± 0.00^b^	0.05 ± 0.00^b^	0.08 ± 0.00^a^
Magnesium (g/100 g)	0.03 ± 0.00^a^	0.04 ± 0.00^a^	0.04 ± 0.00^a^	0.04 ± 0.00^a^
Na (g/100 g)	0.54 ± 0.00^a^	0.18 ± 0.01^b^	0.18 ± 0.00^b^	0.17 ± 0.01^b^
K (g/100 g)	0.16 ± 0.00^b^	0.15 ± 0.00^b^	0.21 ± 0.00^a^	0.22 ± 0.00^a^
Zinc (mg/kg)	30.04 ± 0.65^a^	17.94 ± 0.40^d^	24.92 ± 0.84^b^	20.46 ± 0.53^c^
Iron (mg/kg)	42.82 ± 0.93^d^	83.39 ± 1.88^a^	77.40 ± 2.61^b^	67.26 ± 1.75^c^
Na/K	3.38^a^	1.20^b^	0.86^c^	0.77^d^

*Notes*. Values are means ± standard deviation of three determinations. Values with different superscript on the same row are significant (*p* ≤ 0.05). BCC: 100% Wheat cracker (control); ACC: 100% Acha cracker; APC1: Acha‐pigeon pea cracker (80:20); APC2: Acha‐pigeon pea cracker (70:30); TI: Trypsin Inhibitor.

The presence of antinutrients in food reduces nutrient bioavailability when such food products are consumed. Phytic acid is an antinutrient capable of binding to divalent minerals such as Ca, Mg, Zn, Fe^2+^, hence, affect their absorption. Significantly higher phytate contents (Tables 1) were observed in BCC (1.63 mg/100 g) and ACC (1.36 mg/100 g), and this may be attributed to cereal being the sole source of flour used. Phytates have been reported to be widespread in plant seed grains, including cereals, roots, and tubers (Hidvegi & Lasztity, 2002). APC1 and APC2 had lower phytate contents which suggest that substitution with legume flour reduced the phytate contents of the snacks. The phytate contents of the crackers in this study are lower than those reported for extruded snacks (0.26%) from a blend of pigeon pea and unripe plantain (Anuonye, Jigam, & Ndacek, 2012). Sparvoli et al. (2016) also reported phytate contents ranging from 1.91 to 4.63 mg/g for biscuits made from blends of wheat, maize, and common beans. The oxalate contents of the cereal‐only biscuits (BCC and ACC) showed the lowest (0.09 mg/g) while these contents increased with an increase in legume inclusion, suggesting that pigeon pea seed may have higher oxalate content than cereal (wheat and *acha*). The biscuits can be regarded as safe for consumption without a negative effect on mineral bioavailability (calcium) as the oxalate content is less than the recommended 50–60 mg per day consumption (Chicago Dietetic Association, 2000). The trypsin inhibitor (TI) activity of the *acha*‐pigeon pea biscuits was significantly higher (0.24, 0.28 mg/g) than those of the cereal‐only biscuits (BCC and ACC) which had 0.04 and 0.11 mg/g TI, respectively. TI content was observed to increase with increase in supplementation with pigeon pea flour, which agrees with the report that legumes contain more trypsin inhibitors than cereals (Bunde, Osundahunsi, & Akinoso, 2010). The TI of the formulated snacks is, however, higher than those reported by Olapade et al. (2011) for biscuits from *acha*‐cowpea flours (0.09–0.13 TIU). However, antinutrient contents of the formulated crackers are low, hence high possibility of nutrient (protein, mineral) bioavailability for consumers of the snack.

The composition of calcium, magnesium, iron, and zinc of the crackers ranged from 0.03–0.08 g/100 g, 0.03–0.04 g/100 g, 42.82–83.39 mg/100 g to 17.94–30.04 mg/100 g, respectively. The microelements reduced with an increase in pea supplementation, suggesting that *acha* flour is the main source of mineral in the snacks. The iron contents of the formulated biscuits were significantly higher than those of control (BCC) (*p *<* *0.05). Iron is known to play an important role in nutrition. Potassium has a beneficial effect on sodium balance, and a high dietary intake has been shown to protect human from conditions affecting cardiovascular function. A Na/K ratio less than one is recommended in the diets for regulating blood pressure. The Na/K ranged between 0.77 and 3.38; however, the ratio for APC1 and APC2 was <1; hence, its consumption will not contribute to the sodium level of the consumer and suggests that the biscuits could be suitable snacks for hypertensive and diabetic patients. Healthy biscuits with low Na/K ratio of about 0.5 were reported by Mousa (2014) for wheat‐germinated lupin seed.

### 3.2 Amino acid profile of crackers

The amino acids composition of crackers from wheat flour, *acha* flour, and *acha*‐pigeon pea flour blends are presented in Table 2. Anjum, Ahmad, Butt, Sheikh, and Pasha (2005) reported that cereal proteins are deficient in certain essential amino acids, mainly lysine. Likewise, Sai‐Ut, Ketnawa, Chaiwut, and Rawdkuen (2009) reported that legumes contain adequate amount of lysine though deficient in methionine. Hence, it is necessary to supplement cereal protein with legume protein for a balance of amino acids composition in food products. Anuonye, Onuh, Egwim, and Adeyemo (2010) reported that *acha* grain has the highest methionine content among the cereals. The present study revealed that the use of *acha* and pigeon pea flours for crackers production improved the amino acid composition, especially essential amino acids such as lysine and arginine (62.13% and 30.84% increase, respectively). Results obtained showed that the snacks are very important as protein sources due to the presence of sulfur‐containing amino acids, especially methionine ranging between 1.62 and 4.96 g/100 g with 100% *acha* cracker (ACC) having the highest. The formulated snack also exhibited increased lysine content with a supply of balanced amino acid superior to market sample which served as control (BCC). The biscuits showed a good profile of essential amino acids, which exceeded the FAO/WHO (2007) standards for both adults and children. Most of the amino acids increased with increase in supplementation with pigeon pea flour. The total hydrophobic (HAA) and aromatic amino acids (AAA) of the formulated biscuits were higher than the control. This suggests that ACC, APC1, and APC2 may exhibit better antioxidative properties as AAA are known to freely donate hydrogen atom to electron deficient free radicals, hence, neutralizing the radical as well as breaking the radical chain. Table 3 shows the estimation of nutritional quality of the biscuits. The formulated biscuits (ACC, APC1, and APC2) showed better nutritional qualities than the control (BCC) produced with only wheat flour. Results from nutritional evaluation estimates showed that APC1 and APC2 can contribute 70 and 73% essential amino acids, respectively, compared to the egg's (a complete protein food) nutritional quality. The formulated biscuits were estimated to be capable of supplying 88, 66%, and 85, 63% predicted protein efficiency ratio (P‐PER) and Biological Value (BV), respectively, compared to egg's nutritional quality (USDEC, 1999). P‐PER and BV are important parameters used in evaluating the nutritional significance of protein‐rich foods. BV captures how readily a digested protein can be absorbed and utilized for protein synthesis in human cell. The formulated biscuits are expected to serve as snacks which may accompany some other meal; thus, it suffices to deduce that the formulated biscuit is a nutritionally potent and rich snack that can contribute part of human's daily dietary requirements.

**Table 2:** Amino acid composition of crackers

AA/Samples		Amino acid composition (g/100 g)			FAO/WHO (2007)
	BCC	ACC	APC1	APC2	
Aspartic acid	4.75	7.30	9.28	9.51	
Threonine	2.73	3.80	3.97	4.07	2.3
Serine	5.15	5.25	5.53	5.62	
Glutamic acid	34.58	21.34	20.32	20.05	
Proline	11.53	8.23	7.15	7.01	
Glycine	3.60	2.52	3.07	3.33	
Alanine	2.95	8.83	6.94	6.51	
Cysteine	2.11	2.01	1.62	1.50	0.6
Valine	4.28	5.25	4.95	4.94	3.9
Methionine	1.62	4.96	3.41	2.88	1.6
Isoleucine	3.32	3.54	3.53	3.65	3.0
Leucine	6.71	10.25	9.23	9.02	5.9
Tyrosine	2.79	2.87	2.76	2.74	
Phenylalanine	4.91	5.27	6.20	6.58	3.8
Histidine	2.39	2.85	3.71	4.27	1.5
Lysine	1.83	1.14	3.01	3.08	4.5
Arginine	3.81	2.8 7	4.15	4.15	
Tryptophan	0.97	1.76	1.10	1.10	0.6
HAA	41.18	52.97	46.89	45.93	

*Notes*. BCC: 100% wheat cracker control; ACC: 100% Acha cracker; APC1: Acha‐pigeon pea cracker (80:20); APC2: Acha‐pigeon pea cracker (70:30); FAO: Food and Agriculture Organization; WHO: World Health Organization; HAA: Hydrophobic Amino Acid.

**Table 3:** Estimation of protein quality of formulated crackers

	BCC	ACC	APC1	APC2	REF (Egg) (USDEC, 1999)
TEAA	30.48	40.80	40.80	41.08	57.30
TNEAA	69.52	59.20	59.20	58.92	
EAA/NEAA	0.44	0.69	0.69	0.70	
TSAA	7.37	8.13	8.96	9.31	9.10
TArAA	3.68	6.97	5.03	4.38	5.00
P‐PER	2.21	3.88	3.43	3.34	3.90
BV	23.48	36.27	66.17	63.39	100
CHEMICAL SCORE	36.49	45.88	47.63	46.11	
1^ST^ LIMITING A.A.	Lysine	Histidine	Lysine	Lysine	
2ND LIMITING A.A.	Valine	Lysine	Tryptophan	Isoleucine	

*Notes*. A.A: Amino Acid; TEAA: Total Essential Amino Acid; TNEAA: Total Non‐Essential Amino Acid; TSAA: Total Sulfur Amino Acids; TArAA: Total Aromatic Amino Acids; P‐PER: Predicted Protein Efficiency Ratio; BV: Biological Value; BCC: Control 100% wheat flour cracker; ACC: 100% Acha cracker; APC1: Acha‐pigeon pea cracker (80:20); APC2: Acha‐pigeon pea cracker (70:30), USDEC: United State Dairy Export Council.

### 3.3 Antioxidant properties of formulated crackers

**Figure 1:**
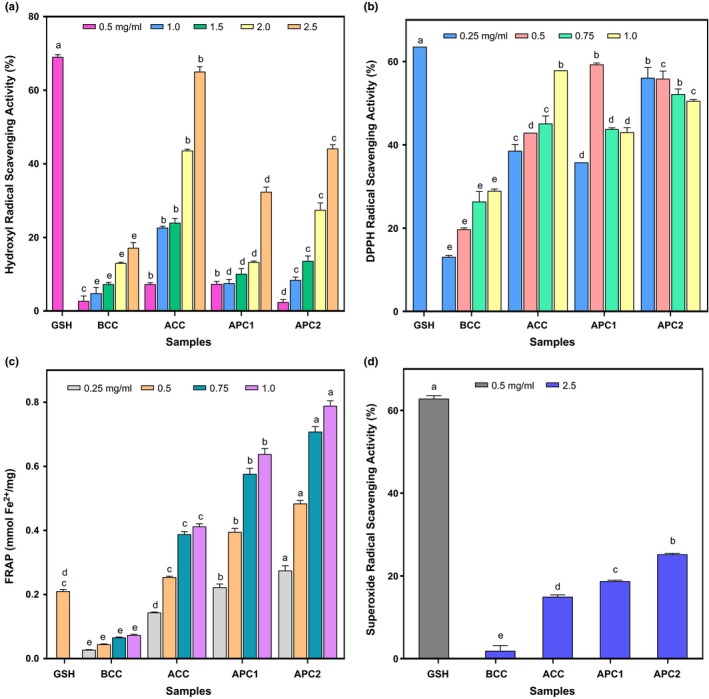
Antioxidant activity of formulated snack. Values are means ± standard deviation of three determinations. Bars with different superscript are significantly different (*p* ≤ 0.05). (a) Percentage hydroxyl radical scavenging activity of crackers; (b) Percentage DPPH radical scavenging activity; (c) Ferric reducing Antioxidant Power in mmol Fe^2+^/mg; (d) Percentage superoxide radical scavenging activity of crackers; GSH: Glutathione; BCC: Control (100% wheat cracker); ACC: 100% Acha cracker; APC1: Acha‐pigeon pea cracker (80:20); APC2: Acha‐pigeon pea cracker (70:30)

Legumes are noted for their high levels of bioactive compounds which can influence glucose metabolism. Incorporation of legume (pigeon pea) flour into cereal flour for biscuits formulation can contribute to the bioactivity of the biscuits. Different mechanisms such as free radical scavenging activities and reducing properties are used by food to exert its antioxidant properties. Free radicals are generated by aerobic organisms during normal metabolic processes. They are highly reactive; oxidize protein, lipids, DNA, and consequently result in oxidative damage. Antioxidants play vital role in defending the human body against generated free radicals. The results of antioxidants activities of the biscuits are presented in Figure 1. The cracker biscuits were evaluated for hydroxyl, DPPH, superoxide radical scavenging activity, and ferric reducing antioxidant power (FRAP). The free radical scavenging activities of the biscuit extracts were concentration dependent for the antioxidant properties evaluated. The results of the hydroxyl radical scavenging activity as shown in Figure 1a ranged from 17.21% to 65.09% with ACC exhibiting significantly the highest ability to inhibit the hydroxyl radical. Although APC1 (32.24%) and APC2 (44.21%) also exhibited high ^•^OH scavenging activity, the biscuits may serve as practical hydroxyl radical scavenger. DPPH^•^ is a stable free radical commonly used in evaluating antioxidant activities of materials within a short time. DPPH^•^ scavenging activity as shown in Figure 1b ranged from 28.96% to 59.34%. APC1 exhibited the highest ability to inhibit the DPPH^•^. ACC (42.94%) and APC2 (55.92%) also exhibited significantly high radical scavenging activities. The formulated biscuits (ACC, APC1, and APC2) were observed to scavenge the DPPH^•^ better than market sample (BCC). The result suggests that they may easily donate free hydrogen atoms to electron deficient DPPH^•^ thereby terminating the radical chain reaction and produce less harmful or unharmful products. The results obtained in the present study are higher than those reported by Adefegha and Oboh (2013) for wheat–bambara biscuits. The superoxide radical scavenging activity (1.91–25.23%) revealed that APC2 exhibited the highest inhibitory activity. The result suggests that the biscuits possess an average potency to scavenge the superoxide radicals.

Ferric Reducing Antioxidant Power (FRAP) is based on the reduction of Fe^3+^ (ferricyanide complex) to Fe^2+^ in the presence of reductants (antioxidants). The Fe^2+^ was monitored by measuring the formation of Perl's Prussian blue at 700 nm (Ferreira, Baptista, Vilas‐Boas, & Barros, 2007). The FRAP value was concentration dependent and ranged from 0.07 to 0.79 mmol Fe^2+^/mg, a significant variation in reducing activity of the biscuit extracts was observed and the pigeon pea supplemented biscuits (APC1 and APC2) exhibited higher FRAP values than non‐supplemented biscuits (BCC and ACC). The ability to reduce Fe^3+^ to Fe^2+^ by the cracker biscuits increased with increase in supplementation with pigeon pea flour which may be attributed to the ability of incorporated pigeon pea flour to form reductants that could react with the free radicals thereby stabilizing and terminating the radical chain (Moktan, Roy, & Sarkar, 2011). The significantly (*p *<* *0.05) higher antioxidant properties observed in the formulated biscuits compare with market sample (BCC) may be attributed to the cereal choice as well as legume flour incorporation which contributed potential bioactive properties to the snacks. Overall, the results of the antioxidant activities of the cracker biscuits suggest that the formulated snacks may be practical radical scavengers, capable of combating key degenerative diseases associated with free radicals as well serving as a functional snack for dietary intervention.

### 3.4 Inhibition of α‐amylase and α‐glucosidase activities

**Figure 2:**
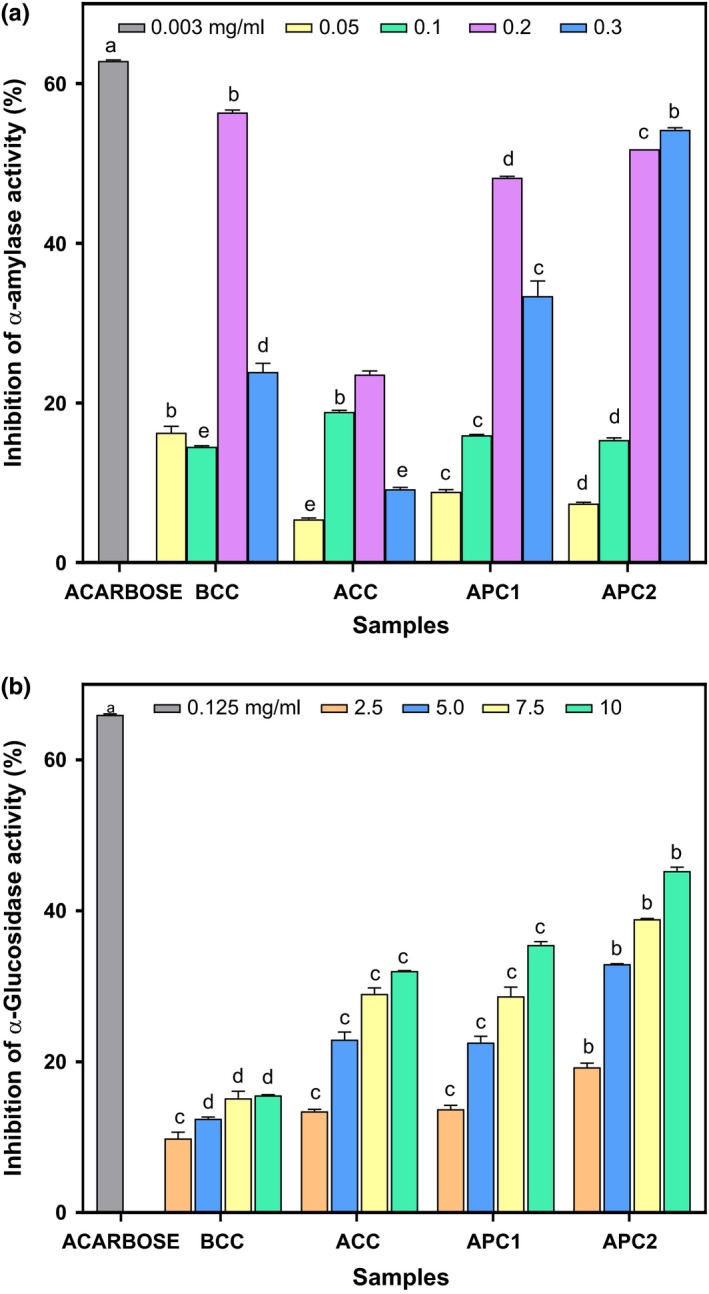
(a) Percentage α‐amylase inhibition of formulated crackers biscuit. Values are means ± standard deviation of three determinations. Bars with different superscript are significantly different (*p* ≤ 0.05). BCC: Control (100% wheat cracker); ACC: 100% Acha cracker; APC1: Acha‐pigeon pea cracker (80:20); APC2: Acha‐pigeon pea cracker (70:30). (b) Percentage α‐glucosidase inhibition of formulated crackers biscuit. Values are means ± standard deviation of three determinations. Bars with different superscript are significantly different (*p* ≤ 0.05). BCC: Control (100% wheat cracker); ACC: 100% Acha cracker; APC1: Acha‐pigeon pea cracker (80:20); APC2: Acha‐pigeon pea cracker (70:30)

The percentage α‐amylase inhibition of *acha*‐pigeon pea cracker is presented in Figure 2a. The result depicts that percentage inhibition was concentration dependent. However, there was a decline in activity at concentrations beyond 0.2 mg/ml except for APC2 which showed progressive increase in activity with increased concentration. ACC (100% *acha* cracker) had the least inhibitory activity (23.59%). Percentage α‐amylase inhibition increased with increase in supplementation with pigeon pea flour with APC1 showing inhibitory activity of 48.26% while APC2 exhibited the highest α‐amylase inhibitory activity (54.22%). There was no significant difference between the percentage α‐amylase inhibitory activities of BCC and APC2. The results obtained are, however, lower than that of acarbose (62.86% at 0.003 mg/ml), a potent antidiabetic drug for the treatment of type II diabetes. This discovery in this study agrees with previous reports which ascertained that plant phytochemicals and underutilized legumes inhibited salivary and pancreatic α‐amylase activities (Ademiluyi & Oboh, 2012; Nickavar & Yousefian, 2009). The high percentage of α‐amylase inhibition may help slow down the absorption of carbohydrates after food intake. The supplementation of the biscuits with legume (proteinaceous α‐amylase inhibitors) may be responsible for the good digestive enzyme inhibitory activity of the biscuits. The percentage α‐glucosidase inhibition of *acha*‐pigeon pea cracker is presented in Figure 2b. The results depict that percentage inhibition was concentration dependent as activities increased with increase in concentration of biscuit extracts, and higher concentration (10 mg/ml) was required to achieve significant inhibitory activity. Contrary to the result of α‐amylase inhibition, control (BCC) showed the least α‐glucosidase inhibitory activity (15.56%), enzyme inhibition increased with increase in supplementation with pigeon pea flour. ACC (32.04%), APC1, and APC2 (35.48% and 45.28% respectively) showed significant α‐glucosidase inhibition. Consequently, APC2 showed the highest α‐glucosidase inhibition potential. Ademiluyi and Oboh (2012) reported a high α‐glucosidase inhibition activity in some legumes studied. Their study also speculated that plant‐based α‐amylase and α‐glucosidase may help lower postprandial hyperglycemia by partially inhibiting the enzymatic hydrolysis of complex carbohydrate which may delay the rapid absorption of glucose. The supplementation of the biscuits with legume (proteinaceous α‐amylase inhibitors) may be responsible for the good digestive enzyme inhibitory activity of the biscuits. The inhibition of α‐amylase will slow down the breakdown of starch to disaccharide while the inhibition of α‐glucosidase will slow down the breakdown of disaccharide to monosaccharide (glucose), thus reducing the amount of glucose absorbed into the blood stream (Ibrahim, Koorbanally, & Islam, 2016). This revealed that both enzymes have a synergistic effect on blood glucose. As the snacks have high inhibitory activity of the two key digestive enzymes, it may be potential products for the management of diabetes.

### 3.5 In vitro protein digestibility and estimated glycemic index of formulated crackers

In vitro protein digestibility (*IV*PD) refers to the actual amount of protein absorbed into the body relative to the amount that was ingested or consumed (Lean, 2007). *IV*PD assessment is carried out to evaluate nutrient bioavailability as nutrient composition (protein content) is not sufficient to predict its bioavailability upon consumption. The protein digestibility profile of the formulated biscuits is presented in Table 4. *IV*PD of the crackers decreased with increase in substitution with pigeon pea flour. The formulated crackers (ACC) with the least protein content (10.47%) had the highest *IV*PD (78.67%) while the crackers with the highest protein content (19.18%) showed the lowest *IV*PD (71.43%). There was no significant difference in the *IV*PD of BCC (72.34%) which had the least protein content (9.32%) and APC2 (71.43%) that showed the highest protein content (19.18%). This could be as a result of the high content of phytate in BCC which may bind to protein thus reducing nutrient availability, digestibility, and utilization. Also, the result suggests that high protein content does not directly imply high digestibility of the protein when ingested. The presence of antinutrients such as trypsin inhibitors has been reported to cause a reduction in protein digestibility, although the amount of trypsin inhibitors in the biscuit samples was quite low to account for the consequently observed decrease in *IV*PD of cracker biscuits containing pigeon pea flour. The observed decrease in *IV*PD may be as a result of nonenzymic browning reaction between flour protein and sugars resulting in possible nonreversible formation of compounds leading to reduction in the protein available for digestion. The results of Ayo, Ayo, Nkama, and Adewori (2007) corroborate the findings in this study. They also observed a reduction in *IV*PD with increase in level of soybean flour in cookies produced from *acha*, wheat, and soybean flours. The values reported in this study are high than those reported by Okpala and Chinyelu (2011) for cocoyam‐pigeon pea (80:20%; 70: 30%) cookies (64.81%; 64.77%, respectively). The results, however, negate the findings of Chinma et al. (2012) who reported increased protein digestibility of cookies upon supplementation with defatted sesame flour.

**Table 4:** In *vitro* protein digestibility and estimated Glycemic Indices of formulated cracker biscuit

	IVPD (%)	eGI (%)	HI (%)
BCC	72.34 ± 0.18^c^	69.73 ± 0.62^a^	54.68 ± 0.06^a^
ACC	78.67 ± 0.27^a^	65.24 ± 0.21^b^	47.41 ± 0.30^b^
APC1	74.87 ± 0.09^b^	55.80 ± 0.16^c^	30.22 ± 0.09^c^
APC2	71.43 ± 0.45^c^	47.95 ± 0.02^d^	15.01 ± 0.41^d^

*Notes*. Values are means ± standard deviation of three determinations. Values with different superscript on the same column are significantly different (*p* ≤ 0.05). BCC: Control 100% Wheat Cracker; ACC: 100% Acha cracker; APC1: Acha‐pigeon pea cracker (80:20); APC2: Acha‐pigeon pea cracker (70:30).

The glycemic index (GI) of the crackers ranged from 47.95% to 69.73%. According to GI classification of foods, values <55 are low GI foods, >55 but <70 are intermediate GI foods and >70 are high GI foods (Barrett & Udani, 2011). APC2 showed low GI (47.95%) while ACC and APC1 (65.24% and 55.80%) exhibited intermediate GI, whereas the control showed high GI. This may be attributed to the raw material choice and nutritional composition of the individual product. According to the classification of food glycemic index (GI), these products (ACC, APC1, APC2) can be considered as low glycemic foods because their GI values are less than 70% (Allen, Corbitt, Maloney, Butt, & Truong, 2012) while BCC is approximately 70% indicating that the market sample is a high glycemic snack. Results obtained showed that as the level of supplementation with pigeon pea flour increased, the GI decreased. Mlotha, Mwangwela, Kasapila, Siyane, and Masamba (2015) reported that low GI food releases glucose more slowly and steadily thus producing more suitable postprandial blood glucose levels. However, foods with high glycemic index (GI) produce a higher peak in postprandial blood glucose and a greater overall blood glucose response during the first 2 h after consumption than foods with low GI. Reduction of GI of a meal can be achieved by inclusion of resistant starches which act like dietary fiber and resist digestion in the small intestine (Englyst & Englyst, 2005). These resistant starches have been reported to be found in seeds, legumes, unprocessed whole grains, a category in which the raw materials used for this study falls into. Oboh, Osagie, and Omotosho (2010) equally reported that pigeon pea seeds possessed low GI. Consumption of foods with low glycemic index value is associated with better health. Thus, the formulated biscuits may serve as functional dietary snacks, specially designed for targeted groups that require low glycemic index foods intake.

## 4 CONCLUSIONS

Increased supplementation of *acha* flour with pigeon pea flour in the flour blend used for crackers in this study resulted in increased protein content and amino acid profile of snacks. The formulated snack also exhibited significant antioxidative properties. *Acha*‐pigeon pea cracker (70:30) showed the highest inhibitory activity against potent digestive enzymes (α‐amylase and α‐glucosidase) responsible for breakdown and absorption of carbohydrate. It equally exhibited the lowest glycemic index which may be relevant in lowering postprandial hyperglycemia. From the foregoing, the formulated crackers especially APC2 may be potential snacks for management of hyperglycemia and may serve as functional foods for the prevention of degenerative diseases.

## ETHICAL STATEMENT

The study did not involve human and animal testing.

## CONFLICT OF I NTEREST

The authors declare that there is no conflict of interests.
